# Varying apparent metabolizable energy concentrations and protease supplementation affected broiler performance and jejunal and ileal nutrient digestibility from 1 to 35 d of age

**DOI:** 10.1016/j.psj.2022.101911

**Published:** 2022-04-08

**Authors:** K.W. McCafferty, N.K. Morgan, A.J. Cowieson, M. Choct, A.F. Moss

**Affiliations:** ⁎ USDA-ARS Poultry Research Unit, MS, 39762, United States; # School of Environmental and Rural Sciences, University of New England, Armidale, NSW, 2351, Australia; § DSM Nutritional Products, Kaiseraugst, 4303, Switzerland

**Keywords:** apparent metabolizable energy, protease, performance, nutrient digestibility, broiler

## Abstract

An experiment was conducted to evaluate the effects of varying AME concentrations and protease supplementation on broiler performance and jejunal and ileal nutrient digestibility from 1 to 35 d of age. Ross × Ross 308 male broilers (n = 1,008) were equally distributed into 48 floor pens and offered 1 of 6 dietary treatments (8 replicate pens/treatment). Dietary treatments consisted of a factorial arrangement with AME concentration (low-, moderate-, or high-AME) and supplemental protease (without or with) as the main factors. Birds and feed were weighed on 1, 15, 29, and 35 d of age to determine body BW, BW gain (**BWG**), feed intake (**FI**) and feed conversion ratio (**FCR**). At 15 and 29 d of age, jejunal and ileal digesta contents were collected to determine nutrient digestibility. From 1 to 15 d of age, broilers offered moderate-AME diets (*P* < 0.05) had 6.7, 7.1, 4.8% higher BW, BWG, FI, respectively, and a 2.1% lower FCR compared with those offered low-AME diets. Likewise, protease increased (*P* < 0.05) BW and BWG by 4.3 and 4.7%, respectively, and decreased (*P* < 0.05) FCR by 3.4%, compared with those offered the diets without protease. From 1 to 29 d of age, broilers offered high-AME diets had 2.9% lower (*P* < 0.05) FCR compared with those offered low-AME diets. Protease increased (*P* < 0.05) BW, BWG, and FI by 3.1, 3.2, and 4.2%, respectively, compared with the unsupplemented diets. From 1 to 35 d of age, broilers receiving high-AME diets had 2.9% lower (*P* < 0.05) FCR compared with those offered low-AME diets. Protease increased (*P* < 0.05) FCR by 1.0% compared with those offered unsupplemented diets. Jejunal (15 and 29 d of age) and ileal (29 d of age) starch digestibility and jejunal nitrogen digestibility (29 d of age) were lower (*P* < 0.05) in broilers offered high-AME diets compared with those offered low-AME diets. Both AME concentration and supplemental protease independently affected broiler performance, with responses being most apparent during early growth phases whereas digestibility measures were mainly influenced by AME concentration.

## INTRODUCTION

The benefits of mono-component protease supplementation in broiler diets have primarily been attributed to improvements in protein and amino acid (**AA**) digestibility (Angel et al., 2011; Cowieson and Roos, 2013; Cowieson and Roos, 2016). Overall, this deduction is quite appropriate, but additional improvements observed in AME and net energy with supplemental protease indicate that the value of protease inclusion may extend beyond improvements in AA digestibility alone (Cowieson et al., 2019). A number of studies have evaluated the effects of protease on AME in broilers, and on average improvements of 80 kcal/kg have been observed (Fru-Nji et al., 2011; Kalmendal and Tauson, 2012; Olukosi et al., 2015; Cowieson et al., 2017a). These improvements in AME are typically greater than the sum of energy contributed by AA digestibility, indicating an improvement in energy partitioning (Cowieson et al., 2019).

Capitalizing on these energy-sparing effects with supplemental protease has potential to improve commercial value and reduce diet costs if results are consistent and quantifiable. Currently, these energy-sparing effects with protease are generally not applied in least-cost feed formulation due to limited mechanistic understanding (Cowieson et al., 2019). Additionally, ingredient variation, nutrient safety margins, and lack of clarity on enzyme additivity with other exogenous enzymes may increase hesitancy to apply an energy matrix value with protease. Therefore, the objective of this experiment was to evaluate the effects of varying AME concentrations (low-, moderate-, or high-AME) and protease supplementation (without or with), in conjunction with phytase and xylanase, on broiler growth performance and jejunal and ileal digestibility of broilers during a 5-wk production period. The hypothesis of this study was that AME concentration and supplemental protease would interact to affect broiler growth performance and nutrient digestibility, and the effects of supplemental protease would be more pronounced in diets with low- and moderate-AME concentrations.

## MATERIALS AND METHODS

### Husbandry and Diets

This experiment was approved by the University of New England Animal Ethics Committee (AEC 19-123), which complies with the Australian Code of Practice for the Care and Use of Animal for Scientific Purposes. One thousand and eight Ross × Ross 308 (Aviagen, Goulburn, NSW, AU) male chicks vaccinated for Marek's disease, Newcastle disease, and infectious bronchitis, were obtained from a commercial hatchery at day of hatch. Chicks were randomly distributed into 48 floor pens (21 chicks per pen; 0.07 m^2^ per bird) across 2 environmentally controlled rooms. Each pen was equipped with fresh wood shavings, a hanging pan-feeder, and lubing drinker cups. Access to feed and water was provided ad libitum throughout the experiment. Room temperature was initially set at 34.0°C at placement and was gradually reduced as the birds advanced in age, with a final set point of 24°C at 35 d of age. Photoperiod was set at 23L: 1D from 1 to 6 d of age, and then 20L: 4D from 7 to 35 d of age.

Prior to formulation, dietary ingredients (corn and soybean meal) were analyzed by near-infrared spectroscopy to predict proximates, AA concentrations, and AME, using AMINONIRPROX, AMINONIRNIR, and AMINONIRNRG (Evonik Nutrition & Care, Hanua, DE), respectively. Six dietary treatments were provided throughout the starter (1–15 d of age; Table 1), grower (16–29 d of age; Table 2), and finisher (30–35 d of age; Table 3) phases. Dietary treatments were arranged in a factorial manner with 3 AME concentrations (low-, moderate-, and high-AME) and 2 protease supplementation levels (without and with) as the main factors. All dietary treatments were formulated to be adequate in essential nutrients, except for digestible AA and AME concentrations. All diets were formulated on a dAA to digestible Lys ratio, but digestible AA concentrations were 11.2 % below primary breeder guidelines (Ross 308 Broiler Nutrient Specifications, 2014). Likewise, low- and high-AME diets were 57 kcal/kg below and above the primary breeder recommendations, respectively, to achieve varying concentrations of AME (Ross 308 Broiler Nutrient Specifications, 2014). All diets contained a mono-component phytase (RONOZYMEHIPhos GT; 10,000 FYT/g) and xylanase (RONOZYMEWX CT; 1,000 FXU/g), expressed by strains of *Aspergillus oryzae*, to achieve feed activity concentrations of 2,000 FYT/kg and 200 FXU/kg, respectively (DSM Nutritional Products, Kaiseraugst, CH). In diet formulation, phytase was anticipated to provide 0.15% of both Ca and P, but no matrix value was applied to xylanase. A mono-component serine protease (RONOZYMEProAct CT; 75,000 PROT/g; DSM Nutritional Products, Kaiseraugst, CH) expressed by *Bacillus licheniformis* was included in the supplemented treatments, to achieve feed activity concentration of 15,000 PROT/kg. No matrix value was applied to supplemental protease. Basal diets were formulated with 0.03% Washed Builder's Sand, and protease was included in the supplemented treatments at its expense (e.g., 0.02% protease + 0.01% sand). These methods of enzyme application (matrix value for phytase and on-top application for xylanase and protease) were designed to mimic those outlined by Cowieson et al. (2019). Diets were cold-pelleted (65°C) and feed form consisted of crumbles during the starter period and pellets thereafter. A commercial laboratory determined the phytase, xylanase, and protease activity concentrations of all experimental treatments (DSM Nutritional Products Australia Pty Ltd, Wagga Wagga, AU).

**Table 1:** Ingredient and nutrient composition of basal starter diets fed to Ross × Ross 308 male broilers from 1 to 15 d of age.

	AME		
Ingredient, % “as-fed”	Low	Moderate	High
Corn	64.41	63.30	62.18
Soybean meal	30.45	30.57	30.69
Canola oil	1.03	2.02	3.00
Calcium carbonate	0.88	0.88	0.88
Dicalcium phosphate	1.11	1.11	1.11
Sodium chloride	0.20	0.20	0.20
Sodium bicarbonate	0.17	0.17	0.17
Vitamin Premix^1^	0.09	0.09	0.09
Mineral Premix^2^	0.10	0.10	0.10
Choline chloride	0.38	0.38	0.38
L-Lys•HCl	0.26	0.26	0.26
DL-Met	0.28	0.28	0.28
L-Thr	0.09	0.09	0.09
Phytase^3^	0.02	0.02	0.02
Xylanase^3^	0.02	0.02	0.02
Sand^4^	0.03	0.03	0.03
Titanium dioxide	0.50	0.50	0.50
Calculated Nutrient Content (%, unless otherwise indicated)			
AME, kcal/kg	2,950	3,007	3,064
Starch	42.54	41.81	41.08
Crude Protein	19.74	19.71	19.67
Digestible Lys	1.11	1.11	1.11
Digestible Met	0.54	0.55	0.55
Digestible Met + Cys	0.81	0.81	0.81
Digestible Thr	0.71	0.71	0.71
Digestible Val	0.82	0.82	0.82
Digestible Arg	1.14	1.14	1.14
Digestible Trp	0.20	0.20	0.20
Ca	0.85	0.85	0.85
Non-phytate P	0.46	0.46	0.46
Na	0.18	0.18	0.18

1 Vitamin premix supplied per kilogram of diet: retinol, 12000 IU; cholecalciferol, 5000 IU; tocopheryl acetate, 75 mg, menadione, 3 mg; thiamine, 3 mg; riboflavin, 8 mg; niacin, 55 mg; pantothenate, 13 mg; pyridoxine, 5 mg; folate, 2 mg; cyanocobalamin, 16 μg; biotin, 200 μg; cereal-based carrier, 149 mg; mineral oil, 2.5 mg.

2 Trace mineral premix supplied per kilogram of diet: Cu (sulphate), 16 mg; Fe (sulphate), 40 mg; I (iodide), 1.25 mg; Se (selenate), 0.3 mg; Mn (sulphate and oxide), 120 mg; Zn (sulphate and oxide), 100 mg; cereal-based carrier, 128 mg; mineral oil, 3.75 mg

3 RONOZYME HiPhos and RONOZYME WX were used as sources of phytase and xylanase, respectively. A phytase matrix value of 0.15% Ca and 0.15% digestible P was used. No energy matrix was applied to xylanase.

4 ProAct was included at 0.00 or 0.02% at the expense of sand to achieve protease activity concentrations of 0 or 15,000 PROT/kg, respectively, in each of the 3 basal diets.

**Table 2:** Ingredient and nutrient composition of basal grower diets fed to Ross × Ross 308 male broilers from 16 to 29 d of age.

	AME		
Ingredient, % “as-fed”	Low	Moderate	High
Corn	66.48	65.37	64.26
Soybean meal	27.56	27.68	27.80
Canola oil	2.15	3.13	4.12
Calcium carbonate	0.91	0.91	0.91
Dicalcium phosphate	0.86	0.86	0.86
Sodium chloride	0.21	0.21	0.21
Sodium bicarbonate	0.17	0.17	0.17
Vitamin Premix^1^	0.09	0.09	0.09
Mineral Premix^2^	0.10	0.10	0.10
Choline chloride	0.36	0.36	0.36
L-Lys•HCl	0.24	0.23	0.23
DL-Met	0.24	0.24	0.24
L-Thr	0.07	0.07	0.07
Phytase^3^	0.02	0.02	0.02
Xylanase^3^	0.02	0.02	0.02
Sand^4^	0.03	0.03	0.03
Titanium dioxide	0.50	0.50	0.50
Calculated Nutrient Content (%, unless otherwise indicated)			
AME, kcal/kg	3,050	3,107	3,164
Starch	43.89	43.16	42.43
Crude Protein	18.61	18.58	18.54
Digestible Lys	1.03	1.03	1.03
Digestible Met	0.66	0.67	0.67
Digestible Met + Cys	0.75	0.75	0.75
Digestible Thr	0.66	0.66	0.66
Digestible Val	0.78	0.78	0.77
Digestible Arg	1.06	1.06	1.06
Digestible Trp	0.19	0.19	0.19
Ca	0.80	0.80	0.80
Non-phytate P	0.41	0.41	0.41
Na	0.18	0.18	0.18

1 Vitamin premix supplied per kilogram of diet: retinol, 12000 IU; cholecalciferol, 5000 IU; tocopheryl acetate, 75 mg, menadione, 3 mg; thiamine, 3 mg; riboflavin, 8 mg; niacin, 55 mg; pantothenate, 13 mg; pyridoxine, 5 mg; folate, 2 mg; cyanocobalamin, 16 μg; biotin, 200 μg; cereal-based carrier, 149 mg; mineral oil, 2.5 mg.

2 Trace mineral premix supplied per kilogram of diet: Cu (sulphate), 16 mg; Fe (sulphate), 40 mg; I (iodide), 1.25 mg; Se (selenate), 0.3 mg; Mn (sulphate and oxide), 120 mg; Zn (sulphate and oxide), 100 mg; cereal-based carrier, 128 mg; mineral oil, 3.75 mg.

3 RONOZYME HiPhos and RONOZYME WX were used as sources of phytase and xylanase respectively. A phytase matrix value of 0.15% Ca and 0.15% digestible P was used. No energy matrix was applied to xylanase.

4 ProAct was included at 0.00 or 0.02% at the expense of sand to achieve protease activity concentrations of 0 or 15,000 PROT/kg, respectively, in each of the 3 basal diets.

**Table 3:** Ingredient and nutrient composition of basal finisher diets fed to Ross × Ross 308 male broilers from 30 to 35 d of age.

	AME		
Ingredient, % “as-fed”	Low	Moderate	High
Corn	69.74	68.62	67.51
Soybean meal	24.13	24.26	24.38
Canola oil	2.58	3.56	4.55
Calcium carbonate	0.92	0.92	0.92
Dicalcium phosphate	0.65	0.65	0.65
Sodium chloride	0.22	0.22	0.22
Sodium bicarbonate	0.18	0.18	0.18
Vitamin Premix^1^	0.09	0.09	0.09
Mineral Premix^2^	0.10	0.10	0.10
Choline chloride	0.35	0.35	0.35
L-Lys•HCl	0.22	0.22	0.22
DL-Met	0.20	0.20	0.20
L-Thr	0.05	0.06	0.06
Phytase^3^	0.02	0.02	0.02
Xylanase^3^	0.02	0.02	0.02
Sand^4^	0.03	0.03	0.03
Titanium dioxide	0.50	0.50	0.50
Calculated Nutrient Content (%, unless otherwise indicated)			
AME, kcal/kg	3,116	3,173	3,230
Starch	46.01	45.28	44.55
Crude Protein	17.27	17.23	17.20
Digestible Lys	0.94	0.94	0.94
Digestible Met	0.59	0.59	0.59
Digestible Met + Cys	0.69	0.69	0.69
Digestible Thr	0.60	0.60	0.60
Digestible Val	0.72	0.72	0.72
Digestible Arg	0.97	0.97	0.97
Digestible Trp	0.17	0.17	0.17
Ca	0.75	0.75	0.75
Non-phytate P	0.37	0.37	0.37
Na	0.18	0.18	0.18

1 Vitamin premix supplied per kilogram of diet: retinol, 12000 IU; cholecalciferol, 5000 IU; tocopheryl acetate, 75 mg, menadione, 3 mg; thiamine, 3 mg; riboflavin, 8 mg; niacin, 55 mg; pantothenate, 13 mg; pyridoxine, 5 mg; folate, 2 mg; cyanocobalamin, 16 μg; biotin, 200 μg; cereal-based carrier, 149 mg; mineral oil, 2.5 mg.

2 Trace mineral premix supplied per kilogram of diet: Cu (sulphate), 16 mg; Fe (sulphate), 40 mg; I (iodide), 1.25 mg; Se (selenate), 0.3 mg; Mn (sulphate and oxide), 120 mg; Zn (sulphate and oxide), 100 mg; cereal-based carrier, 128 mg; mineral oil, 3.75 mg.

3 RONOZYME HiPhos and RONOZYME WX were used as sources of phytase and xylanase respectively. A phytase matrix value of 0.15% Ca and 0.15% digestible P was used. No energy matrix was applied to xylanase.

4 ProAct was included at 0.00 or 0.02% at the expense of sand to achieve protease activity concentrations of 0 or 15,000 PROT/kg, respectively, in each of the 3 basal NC diets.

### Measurements and Calculations

Birds and feed were weighed at 1, 15, 29, and 35 d of age to determine BW, BW gain (**BWG**), feed intake (**FI**), and feed conversion ratio (**FCR**). Mortality was recorded daily and used to adjust FCR on a bird-day basis.

At 15 and 29 d of age, 4 and 3 birds per pen, respectively, were randomly selected, weighed, and euthanized for collection of jejunal and ileal digesta contents. Digesta contents from the entire jejunum (end of duodenal loop to Meckel's diverticulum) and ileum (Meckel's diverticulum to ileo-cecal junction) were gently squeezed into polypropylene cups. Samples were pooled per cage and frozen at –20°C until further analysis. Jejunal and ileal apparent digestibility coefficients (**ADC**) of nitrogen (**N**), starch, and energy were determined using titanium dioxide (**TiO_2_**) as the inert marker and calculated on a DM basis using the following equation:

$$\text{ADC}\;\left(\%\right)\;=\left[\left(\left(\text{Nutrient}/\text{Ti}O_{2}\right)\text{diet}-\left(\text{Nutrient}/\text{Ti}O_{2}\right)\text{digesta}\right)/\left(\text{Nutrient}/\text{Ti}O_{2}\right)\text{diet}\right]$$

where nutrient corresponded to N, starch, or gross energy (**GE**). Also, apparent digestible energy (**ADE**) was calculated using the following equation:

$$\text{ADE}\;\left(\text{kcal}/\text{kg}\right)\;=\;\left[GE_{\text{diet}}\;\times \;\left(\text{ADC}\;\text{of}\;\text{energy}\right)\right].$$

### Chemical Analyses

Diet and digesta samples were freeze dried and ground through a 0.5 mm sieve. Samples were analyzed for nitrogen, starch, and GE. Dry matter of wet samples was determined using a forced air oven (105°C for 12 h). Nitrogen content was determined by the Dumas combustion method (method 990.03; AOAC, 2005) with a Leco FP-200 N analyzer (Leco Corp., St. Joseph, MI), using N correction factor of 6.25 for crude protein (**CP**) determination. Starch concentration was determined enzymatically using Megazyme Total Starch Assay Kit (Megazyme Int., Wicklow Ireland). Gross energy was determined using a 6400 automatic isoperibol oxygen bomb calorimeter (Parr Instruments, Moline, IA) with benzoic acid as the calibration standard. Additionally, diets and digesta were analyzed for TiO_2_ concentrations in quadruplicate and duplicate replicates, respectively, by the method described by (Short et al., 1996).

### Statistical Analysis

The experiment was arranged in a randomized complete block design structure with pen location as the blocking factor, and 8 replicate pens per treatment. Pen was considered as the experimental unit. A two-way ANOVA in PROC MIXED (SAS 9.4, 2015) was used to evaluate the interactive and main effects of AME concentration (low-, moderate-, or high-AME) and protease supplementation (without or with) on growth performance and nutrient digestibility. Statistical significance was established at *P* ≤ 0.05, and a trend was considered at *P* ≤ 0.10. Interactive and main effects were separated using Tukey's Honestly Significantly Different test.

## RESULTS

### Dietary Enzyme Activity

Dietary enzyme activities of phytase, xylanase, and protease are displayed in Table 4. Overall, all analyzed values were within acceptable ranges (<30% difference) compared with calculated values.

**Table 4:** Analyzed activity concentrations of phytase, xylanase, and protease in the starter (1–14 d of age), grower (15–28 d of age), and finisher (29–35 d of age) diets.^1^

	1 to 15 d of age			16 to 29 d of age			30 to 35 d of age		
Dietary treatments^2^	Protease^3^ (PROT/kg)	Xylanase^4^ (FXU/kg)	Phytase^5^ (FYT/kg)	Protease (PROT/kg)	Xylanase (FXU/kg)	Phytase (FYT/kg)	Protease (PROT/kg)	Xylanase (FXU/kg)	Phytase (FYT/kg)
Low-AME without	—6	213	1,983	—	188	1,468	—	237	1,638
Low-AME with	12,790	201	2,082	11,890	206	1,820	10,700	289	1,769
Moderate-AME without	—	231	2,154	—	231	1,645	—	186	1,737
Moderate- AME with	12,230	201	2,440	12,330	230	1,806	11,650	175	2,045
High-AME without	—	230	2,090	—	187	2,028	—	202	2,081
High- AME with	10,990	196	1,962	11,390	233	1,955	13,860	246	1,798

1 Values represent average of 3 replicates samples. Enzyme activity was determined by an outside laboratory (DSM Nutritional Products, Wagga Wagga, NSW, Australia).

2 Dietary treatments consisted of a factorial arrangement with AME concentrations (low- moderate- or high-AME) and protease supplementation (without or with) as the main factors.

3 Protease = RONOZYME ProAct (DSM Nutritional Products, Kaiseraugst, CH) which provides 75,000 PROT/g was included in the supplemental treatments at 0.02% to achieve a protease activity of 15,000 PROT/kg.

4 Xylanase = RONOZYME WX (DSM Nutritional Products, Kaiseraugst, CH) which provides 1,000 FXU/g was included in all supplemented treatments at 0.02% to achieve a xylanase activity of 200 FXU/kg.

5 Phytase = RONOZYME HiPhos (DSM Nutritional Products, Kaiseraugst, CH) which provides 10,000 FYT/g was included in all treatments at 0.02% to achieve a phytase activity of 2,000 FYT/kg.

6 — = not analyzed.

### Growth Performance

From 1 to 15 d of age, AME concentration and protease did not interact to affect broiler growth performance (Table 5). A main effect of AME concentration (*P* < 0.05) affected BW (*P* = 0.001), BWG (*P* = 0.001), FI (*P* = 0.009), and FCR (*P* = 0.01). Broilers fed moderate-AME diets had a 6.7 and 7.1% higher (*P* < 0.05) BW and BWG, respectively, compared with those offered the low-AME diets, whereas broilers offered the high-AME diets presented similar BW and BWG compared to those offered low- and moderate-AME diets. Broilers receiving the moderate-AME diets consumed 4.8 and 4.0% more (*P* < 0.05) feed than those offered the low- and high-AME diets, respectively. Broilers offered the low-AME diet had 2.1 and 2.7% higher (*P* < 0.05) FCR than those offered the moderate- and high-AME diets, respectively. A main effect of supplemental protease (*P* < 0.05) influenced BW (*P* = 0.001), BWG (*P* = 0.001), and FCR (*P* = 0.001). Broilers receiving diets with supplemental protease had 4.3 and 4.7% higher (*P* < 0.05) BW and BWG, respectively, and a 3.4% lower (*P* < 0.05) FCR compared with those fed diets without supplemental protease.

**Table 5:** Growth performance of Ross × Ross 308 male broilers fed diets varying in apparent metabolizable energy (AME) and supplemental protease concentrations from 1 to 15 d of age.^1^

AME	Protease^2^	BW (kg)	BW Gain (kg)	Feed Intake (kg)	FCR^3^ (kg:kg)	Mortality (%)
Low	Without	0.454	0.417	0.500	1.198	2.4
	With	0.467	0.431	0.493	1.146	1.3
Moderate	Without	0.484	0.447	0.518	1.158	2.4
	With	0.499	0.462	0.524	1.136	1.3
High	Without	0.460	0.422	0.491	1.164	1.9
	With	0.493	0.456	0.510	1.118	5.4
SEM		0.007	0.007	0.008	0.011	1.3
AME main effects						
Low		0.461^b^	0.424^b^	0.497^b^	1.172^a^	1.8
Moderate		0.492^a^	0.454^a^	0.521^a^	1.147^b^	1.8
High		0.476^ab^	0.439^ab^	0.501^b^	1.141^b^	3.6
SEM		0.005	0.005	0.006	0.009	0.9
Protease main effects						
	Without	0.466^b^	0.429^b^	0.503	1.173^a^	2.2
	With	0.486^a^	0.449^a^	0.509	1.133^b^	2.6
	SEM	0.004	0.004	0.005	0.008	0.7
*Analysis of Variance*				*Probabilities*^4^		
AME × Protease		0.27	0.27	0.33	0.29	0.12
AME		0.001	0.001	0.009	0.01	0.26
Protease		0.001	0.001	0.35	0.001	0.69

1 Each value represents the least-square means of 8 replicate pens with approximately 21 chicks at placement.

2 Protease = RONOZYME ProAct (DSM Nutritional Products, Kaiseraugst, CH), which provides 75,000 PROT/g was included at 0.02% in the supplemented treatments to achieve an activity of 15,000 PROT/kg.

3 FCR = feed conversion ratio corrected for mortality.

4a-b Means within a column for a given measurement not sharing a common superscript differ (*P* ≤ 0.05) and were separated using Tukey's Honestly Significant Difference test.

From 1 to 29 d of age, no interactive effects between AME concentration and supplemental protease on broiler growth performance were observed (Table 6). However, a main effect of AME concentration (*P* < 0.05) on FCR (*P* = 0.001) was observed, with broilers offered the high-AME diet having 2.9% lower (*P* < 0.05) FCR compared with those offered low-AME diets. FCR of those offered the moderate-AME diets was similar to those offered low- and high-AME diets. A protease main effect (*P* < 0.05) influenced BW (*P* = 0.04), BWG (*P* = 0.04), and FI (*P* = 0.01), with broilers receiving diets with supplemental protease having a 3.1, 3.2, and 4.1% higher BW, BWG, and FI, respectively, than those fed diets without supplemental protease.

**Table 6:** Growth performance of Ross × Ross 308 male broilers fed diets varying in apparent metabolizable energy (AME) and supplemental protease concentrations from 1 to 29 d of age.^1^

AME	Protease^2^	BW (kg)	BW Gain (kg)	Feed Intake (kg)	FCR^3^ (kg:kg)	Mortality (%)
Low	Without	1.568	1.531	2.036	1.331	2.4
	With	1.631	1.595	2.120	1.330	1.3
Moderate	Without	1.647	1.610	2.097	1.303	3.5
	With	1.649	1.612	2.127	1.319	2.5
High	Without	1.584	1.547	1.982	1.282	1.9
	With	1.669	1.632	2.121	1.300	5.4
SEM		0.029	0.029	0.038	0.010	1.4
AME main effects						
Low		1.599	1.563	2.078	1.330^a^	1.8
Moderate		1.648	1.611	2.112	1.311^ab^	3.0
High		1.627	1.589	2.051	1.291^b^	3.6
SEM		0.021	0.021	0.026	0.008	1.0
Protease main effects						
	Without	1.600^b^	1.563^b^	2.038^b^	1.305	2.6
	With	1.650^a^	1.613^a^	2.123^a^	1.316	3.0
	SEM	0.017	0.017	0.022	0.008	0.8
*Analysis of Variance*				*Probabilities*^4^		
AME × Protease		0.36	0.36	0.36	0.52	0.17
AME		0.27	0.27	0.29	0.001	0.41
Protease		0.04	0.04	0.01	0.13	0.68

1 Each value represents the least-square means of 8 replicate pens with approximately 21 chicks at placement.

2 Protease = RONOZYME ProAct (DSM Nutritional Products, Kaiseraugst, CH), which provides 75,000 PROT/g was included at 0.02% in the supplemented treatments to achieve an activity of 15,000 PROT/kg.

3 FCR = feed conversion ratio corrected for mortality.

4a-b Means within a column for a given measurement not sharing a common superscript differ (*P* ≤ 0.05) and were separated using Tukey's Honestly Significant Difference test.

From 1 to 35 d of age, there was no interaction between AME concentration and supplemental protease on broiler growth performance (Table 7). However, as a main effect, AME concentration (*P* < 0.05) influenced FCR (*P* = 0.001), with broilers offered the high-AME diets having a 2.1 and 2.0% lower (*P* < 0.05) FCR than those offered the moderate- and low-AME diets, respectively. Likewise, a main effect of protease supplementation on FCR (*P* = 0.03) was observed, with those offered the diets with supplemental protease having 1.0% higher (*P* < 0.05) FCR than those offered the diets without supplemental protease.

**Table 7:** Growth performance of Ross × Ross 308 male broilers fed diets varying in apparent metabolizable energy (AME) and supplemental protease concentrations from 1 to 35 d of age.^1^

AME	Protease^2^	BW (kg)	BW Gain (kg)	Feed Intake (kg)	FCR^3^ (kg:kg)	Mortality (%)
Low	Without	2.243	2.206	3.056	1.386	2.4
	With	2.306	2.269	3.142	1.385	1.9
Moderate	Without	2.311	2.274	3.130	1.377	3.5
	With	2.272	2.235	3.124	1.398	2.5
High	Without	2.253	2.215	2.983	1.347	2.5
	With	2.327	2.290	3.137	1.370	6.0
SEM		0.038	0.038	0.052	0.008	1.3
AME main effects						
Low		2.274	2.238	3.099	1.385^a^	2.1
Moderate		2.291	2.254	3.127	1.387^a^	3.0
High		2.290	2.253	3.060	1.358^b^	4.3
SEM		0.027	0.027	0.038	0.006	1.0
Protease main effects						
	Without	2.269	2.232	3.056	1.370^b^	2.8
	With	2.302	2.265	3.134	1.384^a^	3.5
	SEM	0.023	0.022	0.031	0.005	0.8
*Analysis of Variance*				*Probabilities*^4^		
AME × Protease		0.27	0.27	0.32	0.25	0.19
AME		0.89	0.89	0.44	0.001	0.29
Protease		0.30	0.30	0.08	0.03	0.54

1 Each value represents the least-square means of 8 replicate pens with approximately 21 chicks at placement.

2 Protease = RONOZYME ProAct (DSM Nutritional Products, Kaiseraugst, CH) which provides 75,000 PROT/g was included at 0.02% in the supplemented treatments to achieve an activity of 15,000 PROT/kg.

3 FCR: feed conversion ratio corrected for mortality.

4a-b Means within a column for a given measurement not sharing a common superscript differ (*P* ≤ 0.05) and were separated using Tukey's Honestly Significant Difference test.

### Jejunal and Ileal Digestibility

At 15 d of age, AME concentration and supplemental protease interactive effects (*P* < 0.05) were observed on jejunal N ADC (*P* = 0.008), jejunal ADE (*P* = 0.01) and ileal ADE (*P* = 0.01; Table 8). Jejunal N ADC in broilers receiving the low-AME diet with supplemental protease and the moderate-AME diet without supplemental protease was 3.4% higher (*P* < 0.05) compared with those receiving the high-AME diet with supplemental protease. However, jejunal N ADC of all other treatments were similar to those offered the low-AME diet with supplemental protease, the moderate-AME diet without supplemental protease, and the high-AME diet with supplemental protease. Jejunal ADE of broilers offered the low-AME diet with supplemental protease was 9.1% higher (*P* < 0.05) than those offered the low-AME diet without supplemental protease. Jejunal ADE of those offered moderate- and high-AME diets without and with supplemental were similar to those offered the low-AME diets without and with supplemental protease. Ileal ADE of broilers receiving the high-AME diet with supplemental protease was 2.8% higher (*P* < 0.05) than those offered the moderate-AME diet with supplemental protease. Ileal ADE of all other treatments were similar to those receiving the high-AME diet with supplemental protease and the moderate-AME diet with supplemental protease. A main effect of AME concentration (*P* < 0.05) on jejunal starch ADC (*P* = 0.008) was observed, with broilers offered the low-AME diet having a 3.5% higher (*P* < 0.05) starch digestibility than those offered high-AME diets. However, the starch ADC of broilers receiving moderate-AME diets was similar to those receiving the low- and high-AME diets.

**Table 8:** Apparent jejunal and ileal nitrogen and starch digestibility and digestible energy of Ross × Ross 308 male broilers fed diets varying in apparent metabolizable energy (AME) supplemental protease concentrations at 15 d of age.^1^

		Nitrogen (%)		Starch (%)		DE^3^ (kcal/kg)	
AME	Protease^2^	Jejunum	Ileum	Jejunum	Ileum	Jejunum	Ileum
Low	Without	68.92^ab^	84.55	80.53	97.11	2,293^b^	3,332^ab^
	With	70.41^a^	83.84	81.73	96.97	2,502^a^	3,356^ab^
Moderate	Without	70.48^a^	84.54	79.51	96.96	2,427^ab^	3,363^ab^
	With	69.44^ab^	83.88	79.77	96.61	2,346^ab^	3,278^b^
High	Without	70.17^ab^	83.42	78.76	96.37	2,432^ab^	3,324^ab^
	With	68.10^b^	83.93	78.01	96.69	2,414^ab^	3,371^a^
SEM		0.77	0.43	0.90	0.25	56	24
AME main effects							
Low		69.67	84.19	81.13^a^	97.04	2,398	3,344
Moderate		70.00	84.21	79.64^ab^	96.79	2,386	3,320
High		69.13	83.67	78.35^b^	96.53	2,423	3,348
SEM		0.67	0.34	0.69	0.19	45	19
Protease main effects							
	Without	69.86	84.17	79.64	96.18	2,384	3,340
	With	69.32	83.88	79.84	96.76	2,421	3,335
	SEM	0.63	0.30	0.60	0.17	41	17
*Analysis of Variance*				*Probabilities*^4^			
AME × Protease		0.008	0.19	0.50	0.35	0.01	0.01
AME		0.33	0.27	0.008	0.11	0.72	0.41
Protease		0.24	0.35	0.72	0.77	0.34	0.79

1 Values represent the least-square means of pooled digesta from 4 birds per pen with 8 replicate pens.

2 Protease = RONOZYME ProAct (DSM Nutritional Products, Kaiseraugst, CH) which provides 75,000 PROT/g was included at 0.02% in the supplemented treatments to achieve an activity of 15,000 PROT/kg.

3 DE: digestible energy.

4a-d Means within a column for a given measurement not sharing a common superscript are different (*P* ≤ 0.05). Least squared means were separated using Tukey's Honestly Significantly Different Test.

At 29 d of age, AME concentration and supplemental protease did not interact to affect jejunal and ileal apparent nutrient digestibility (starch, N, and DE) (Table 9). However, a main effect of AME concentration (*P* < 0.05) was observed for jejunal N (*P* = 0.04), jejunal starch (*P* = 0.005), and ileal starch (*P* = 0.05) digestibility. Broilers receiving low-AME diets had a 3.3% higher (*P* < 0.05) jejunal N digestibility than those offered the high-AME diets, whereas the N digestibility of broilers receiving moderate-AME diets was similar to those offered the low- and high-AME diets. Similarly, jejunal starch digestibility of broilers offered low-AME diets was 5.4 and 4.0% higher (*P* < 0.05) than those offered the moderate- and high-AME diets, respectively. Moreover, ileal starch digestibility in broilers offered low-AME diets was 0.88% higher (*P* < 0.05) than those offered the high-AME diets, but starch digestibility of those receiving moderate-AME diets was similar to those offered low- and high-AME diets.

**Table 9:** Apparent jejunal and ileal nitrogen and starch digestibility and digestible energy of Ross × Ross 308 male broilers fed diets varying in apparent metabolizable energy (AME) supplemental protease concentrations at 29 d of age.^1^

		Nitrogen (%)		Starch (%)		DE^3^ (kcal/kg)	
AME	Protease^2^	Jejunum	Ileum	Jejunum	Ileum	Jejunum	Ileum
Low	Without	71.73	85.98	78.74	96.61	2,604	3,510
	With	71.53	84.96	77.37	96.28	2,656	3,482
Moderate	Without	69.98	84.81	73.95	96.01	2,467	3,468
	With	70.70	85.19	74.20	96.22	2,604	3,532
High	Without	69.29	84.49	74.80	95.75	2,501	3,524
	With	69.42	84.86	75.32	95.46	2,569	3,517
SEM		0.85	0.53	1.16	0.34	60	26
AME Main effects							
Low		71.63^a^	85.47	78.06^a^	96.45^a^	2,629	3,494
Moderate		70.34^ab^	85.00	74.07^b^	96.11^ab^	2,536	3,500
High		69.36^b^	84.67	75.06^b^	95.61^b^	2,535	3,520
SEM		0.60	0.37	0.82	0.24	42	18
Protease main effects							
	Without	70.33	85.09	75.83	96.12	2,524	3,499
	With	70.55	85.01	75.63	96.00	2,610	3,510
	SEM	0.49	0.31	0.67	0.19	35	15
*Analysis of Variance*				*Probabilities*^4^			
AME × Protease		0.86	0.32	0.69	0.67	0.76	0.21
AME		0.04	0.33	0.005	0.05	0.21	0.57
Protease		0.76	0.84	0.84	0.62	0.09	0.59

1 Values represent the least-square means of pooled digesta from 4 birds per pen with 8 replicate pens.

2 Protease = RONOZYME ProAct (DSM Nutritional Products, Kaiseraugst, CH) which provides 75,000 PROT/g was included at 0.02% in the supplemented treatments to achieve an activity of 15,000 PROT/kg.

3 DE: digestible energy.

4a-d Means within a column for a given measurement not sharing a common superscript are different (*P* ≤ 0.05). Least squared means were separated using Tukey's Honestly Significantly Different Test.

## DISCUSSION

The impact of varying AME concentrations and supplemental protease was evaluated in this study. Overall, these results did not demonstrate an interaction between AME concentration and supplemental protease on broiler growth performance, but both AME concentration and protease independently affected broiler growth performance and these responses were most evident from 1 to 15 and 1 to 29 d of age. Reducing AME concentration by 57 kcal/kg had a significant impact on broiler BW (−6.3%), BWG (−6.6%), FI (−4.6%), and FCR (+2.2%) during the starter period. This is in agreement with Niu et al. (2009), who observed a similar response on broiler growth performance when feeding various concentrations of AME (2,942, 2,999, and 3,100 kcal/kg) from 1 to 21 d of age. These authors observed that feeding broilers low-AME diets (2,942 kcal/kg) decreased BW (−5.0%), BWG (−4.7), and FI (−1.8%), and increased FCR (+3.0%) compared with those fed moderate-AME diets (2,999 kcal/kg). This is likely due to physical limitations in young broilers, which limits their ability to compensate for reduced AME concentrations (Griffiths et al., 1977; Hidalgo et al., 2004). Therefore, feeding adequate or higher AME concentrations may be necessary for optimizing early growth performance in broilers. However, in the current study, as the broilers advanced in age the effects of AME concentration were only observed to impact FCR. From 1 to 29 and 1 to 35 d of age, broilers receiving moderate- and high-AME diets exhibited better FCR than those offered the low-AME diets. Therefore, the physical limitations that reduced growth rate and feed intake were mitigated as the broiler advanced in age, but the negative effect on FCR remained unresolved. This underscores the importance of maintaining adequate AME concentrations, especially for broilers marketed at lighter weights (Hidalgo et al., 2004).

Early growth performance was improved by supplemental protease. Protease supplementation increased BW (+4.3%) and BWG (+4.7%) and decreased FCR (−3.4%) in broilers from 1 to 15 d of age. Angel et al. (2011) observed a similar response, with protease supplementation significantly increasing BWG (+6.3%) and numerically reducing FCR (−3.3%) in broilers fed corn-soybean meal diets from 7 to 22 d of age. These authors attributed these performance improvements to positive increases in ileal CP and AA digestibility observed with supplemental protease (Angel et al., 2011). However, in the present study, protease supplementation did not significantly affect jejunal and ileal N digestibility at 15 d of age. This may indicate a mode of action by the protease other than direct digestibility of CP and AA was responsible for the positive effects on broiler growth performance. Cowieson et al. (2019) emphasized the importance of distinguishing between the direct and net effects of exogenous enzymes for understanding and explaining differences in growth rate and feed efficiency. Improvements in net energy (Cowieson et al., 2019) and alterations in endogenous enzyme secretions and digestive organs sizes with exogenous protease and/or amylase supplementation (Mahagna et al., 1995; Jiang et al., 2008; Yin et al., 2018) indicate that these secondary or indirect modes of action are likely contributing to the value of supplementation. Understanding and quantifying these net effects will improve application and help explain the magnitude of responses observed on broiler growth rate and efficiency with protease supplementation. From 1 to 29 d of age, the positive effects of protease supplementation on BW (+3.1%) and BWG (+3.2%) remained apparent, along with an increase in FI (+4.2%). This FI effect with supplemental protease is not readily explainable, but likely caused the FCR effect to no longer be apparent from 1 to 29 d of age. Yu et al. (2007) observed contrasting FI effects when supplementing diets with an enzyme admixture (protease, amylase, and xylanase). These authors observed an increase in FI during the grower phase (+1.9%) and cumulatively (+1.9%) when the enzyme admixture was supplemented in the control diet, whereas a decrease in FI was observed during the grower phase (−1.1%) and cumulatively (1.7%) when the enzyme admixture was supplemented in the low protein AA control diet. These results indicate that FI responses with enzyme supplementation may vary depending on the dietary nutrient content. Regardless, in the current study the increased FI observed with protease supplementation was accompanied by higher BW and BWG and no differences in FCR from 1 to 29 d of age were observed. However, protease supplementation trended (*P* = 0.08) to affect cumulative (1–35 d of age) FI, with broilers consuming more feed with supplementation. This may explain why there was a slight increase in cumulative FCR (+1%) with supplemental protease. However, it is important to note that BW and BWG of broilers receiving supplement protease was numerically higher than those without supplemental protease, and the cumulative BW (+0.8%), BWG (+1.0), FI (−5.6%), and FCR (−6.5%) of the birds in this experiment were better than male broiler performance standards specified by the primary breeder (Ross, 2014). However, additional research exploring the interactive effects of protease supplementation and age on FI in broilers is warranted to better understand this response.

In the present work, differences in nutrient digestibility were rather small, and consistently due to varying AME concentrations. Interactive effects on jejunal N ADC and ADE and ileal ADE were observed at 15 d of age, but the lack of significant main effects with these measures and the lack of a consistent pattern made it difficult to interpret any meaningful results. However, broilers fed the low-AME diet with supplemental protease had a 9.1% higher jejunal ADE than those receiving the same diet without supplemental protease. This supports the hypothesis that protease supplementation increases nutrient digestibility in the proximal regions of the small intestine (Liu et al., 2013). Moreover, protease supplementation in wheat-soybean meal based diets has been observed to reduce taurine concentration in the jejunal digesta (1097 vs. 870 mg/kg) in broilers, which is indicative of reduced bile acid secretion (Cowieson et al., 2017b). Cowieson et al. (2017b) suggested that exogenous protease supplementation indirectly increases the digestion of lipids by disrupting the dietary matrix, which reduces bile acid secretion. Therefore, this improvement in jejunal ADE may be related to a net effect of supplemental protease on bile acid secretion. Additionally, AME concentration affected jejunal (15 and 29 d of age) and ileal (29 d of age) starch ADC and jejunal N ADC (29 d of age). In general, broilers receiving low-AME diets exhibited higher ADC than those offered high-AME diets. This response is likely related to dietary lipid concentrations because increases in AME concentrations typically increases lipid inclusion, which has been observed to reduce intestinal transit time (Larbier et al., 1977). Moreover, other researchers have also demonstrated that dietary lipid inclusion can alter the AME of carbohydrates and increase intestinal residency of feed (Mateos and Sell, 1980a, b; Mateos and Sell, 1981). Therefore, the lower rate of apparent digestibility in the jejunum (N and starch) and ileum (starch) in broilers offered high-AME diets compared with those offered low-AME diets was likely due to a slower intestinal transit time which delayed the movement of the indirect marker. However, the lack of differences in ADE and the improved performance of broilers offered high-AME diets indicate that the higher caloric content provided by the lipids contributed to a more efficient use of dietary nutrients.

In conclusion, this present study demonstrated that both AME concentrations and supplemental protease independently affected broiler performance. Feeding adequate or higher-AME concentrations resulted in optimal early growth performance and cumulative FCR. Protease supplementation positively influenced broiler performance from 1 to 15 and 1 to 29 d of age. The minimal interactive and absent main effects of supplemental protease on nutrient digestibility, in conjunction with its positive effects on performance, indicate that its benefits extend beyond direct digestibility effects to additional net effects. Further research evaluating these net effects and the modes of action behind them is warranted to improve understanding and application.

## Disclosures

None.
